# Enhanced YOLOv8 for industrial polymer films: a semi-supervised framework for micron-scale defect detection

**DOI:** 10.3389/frai.2025.1638772

**Published:** 2025-09-09

**Authors:** Xiaoxia Yu, Bingyu Hu, Weifeng Jiang, Jinru Wan, Xinduoji Yang, Nianbo Liu, Xiaoyan Dong

**Affiliations:** ^1^Zhejiang Juhua Co Ltd., Quzhou, China; ^2^School of Computer Science and Engineering, University of Electronic Science and Technology of China, Chengdu, China; ^3^Yangtze Delta Region Institute(Quzhou), University of Electronic Science and Technology of China, Quzhou, China; ^4^Juhua Group Corporation, Quzhou, China; ^5^Zhejiang Guanwei Intelligent Technology Co., Ltd., Quzhou, China

**Keywords:** micron defect detection with YOLOv8 deep learning, polymer material film, defect detection, YOLOv8 algorithm, CBAM, Mean Teacher, NWD

## Abstract

**Introduction:**

Polymer material films are produced through extrusion machines, and their surfaces can develop micro-defects due to process and operational influences. The quantity and size of these defects significantly impact product quality.

**Methods:**

As traditional machine learning defect detection methods suffer from low accuracy and poor adaptability to complex scenarios, requiring extensive effort for parameter tuning and exhibiting weak generalization capability, this paper proposes an improved YOLOv8 method to identify micro-defects on films. The approach embeds the CBAM attention mechanism into high-level networks to address feature sparsity in small target detection samples. Simultaneously, given the difficulty in obtaining large annotated datasets, we employ the Mean Teacher method for semi-supervised learning using limited labeled data. During training, the method optimizes neural network gradients through an improved loss function based on normalized Wasserstein distance (NWD), mitigating gradient instability caused by scale variations and enhancing detection accuracy for small targets. Additionally, a proposed multi-threshold mask segmentation algorithm extracts defect contours for further feature analysis.

**Results:**

Experimental results demonstrate that the improved YOLOv8 algorithm achieves an 8.26% increase in mAP@0.5 compared to the baseline. It exhibits higher precision for small targets, and maintains defect detection rates exceeding 95.0% across validation data of varying image sizes, thereby meeting industrial production requirements. In generalization validation, the model demonstrates superior performance compared to traditional methods under test environments with lighting variations and environmental contamination.

**Discussion:**

The improved YOLOv8 algorithm meeting the stringent requirements for high-precision small-target defect detection on polymer material film in industrial production. Future work will explore more advanced techniques to enhance model accuracy and robustness.

## 1 Introduction

Composite substrates play a crucial role in industrial development. By combining the characteristics of different materials such as plastics, metals, and paper, composite substrates can significantly enhance the performance of packaging materials. The cohesive properties of composite materials (Hsissou et al., 2021; Chen et al., 2021b) enable them to provide excellent moisture resistance, oxygen barrier properties, and UV protection, ensuring product quality during storage and transportation. Polymer material films, with their superior heat-sealing performance and transparency, serve as one of the primary composite substrates for packaging, used in producing high-temperature retort films, vacuum aluminum-plated films, etc. In modern manufacturing, particularly in the production of high-performance film materials, ensuring product quality and stability is paramount.

As an important film material, polymer films may develop surface defects such as crystalline spots and black dots due to raw material contamination, production processes, or operator errors. These defects (Bhatt et al., 2021) severely affect film quality and negatively impact the performance and market acceptance of final products. Fewer defects result in superior product properties including ductility, transparency, glossiness, and thickness uniformity. Therefore, defect detection for polymer films prior to shipment is essential. However, as film defects are extremely small (micron-scale) and mostly invisible to the naked eye, traditional machine learning methods for defect detection prove time-consuming and inadequate for high-efficiency production line requirements. Consequently, precise and high-performance defect detection systems have become critical in polymer film production. This study focuses on detecting defects in polymer films made from FEP particles. Traditional industrial inspection (Chen et al., 2021a) typically relies on machine learning methods requiring extensive parameter tuning, exhibiting low fault tolerance and detection confidence. Thus, designing more accurate detection processes for polymer film production is essential for quality improvement and will drive advancements in industrial inspection.

Automated inspection systems in industrial environments (Singh and Desai, 2023) leverage advanced sensors, machine vision, and algorithmic technologies to inspect every product on production lines, ensuring timely identification of potential defects or quality issues. Compared to traditional machine learning approaches, deep learning-based automated systems operate at exceptional speeds, enabling 24/7 high-precision detection. These systems also achieve higher consistency and accuracy, reducing defective products and rework while ensuring strict compliance with quality standards. By implementing automated inspection, enterprises can reduce operational costs, enhance production line automation, and promote intelligent manufacturing upgrades. Such technological applications help companies maintain competitive advantages in dynamic markets while laying foundations for future growth and innovation.

The application of object detection models in defect detection holds significant practical value, particularly in industrial production and quality control. Advanced object detection models enable enterprises to achieve precise defect identification (Hussain, 2023). On production lines, these models can systematically identify potential defects, allowing rapid corrective actions. Moreover, their implementation enhances production intelligence by enabling real-time quality monitoring and ensuring pre-shipment compliance with stringent standards. As production scales expand, traditional machine learning methods face increasing time and cost pressures, while deep learning-based object detection models effectively mitigate these issues. Through automated inspection, enterprises strengthen quality control, ultimately enhancing market competitiveness and customer satisfaction.

Industrial inspection of polymer films demands micron-scale defect detection with higher accuracy, yet faces three key challenges: feature sparsity, label scarcity and environmental noise. Compared to general object detection, small-target detection tasks pose distinct challenges due to the sparsity of small objects. According to the COCO dataset standard, small objects are defined as those with a bounding box area smaller than 32 × 32 pixels. In the defect detection scenario for polymer material thin films studied here, defective regions typically span only a few pixels, with diameters ranging from 0 to 800 micrometers. Nevertheless, their importance in safety, quality control, and environmental monitoring remains undeniable. First, high morphological similarity among small targets complicates their differentiation in images. The limited pixel coverage of small targets leads to information loss, hindering effective feature extraction for recognition. This feature scarcity exacerbates detection difficulties. Additionally, small targets are often obscured by complex backgrounds, increasing detection challenges. Background noise and interference from other objects may cause false positives or missed detections, further compromising system reliability. These factors not only degrade model performance but may also lead to misidentification of critical targets, affecting subsequent decision-making. Furthermore, small targets exhibit significant variations in appearance under different distances and viewing angles, demanding detection models with exceptional adaptability and robustness.

To address the low accuracy of traditional industrial machine learning in defect detection, this study proposes an improved YOLOv8 method for polymer film defect detection. The approach utilizes advanced object detection technology to automate defect recognition through image acquisition and analysis of film surfaces. During defect detection, the system performs real-time identification of various defects through feature extraction and global information integration of acquired data. In subsequent statistical analysis, a hierarchical detection method combining multi-threshold segmentation, morphological analysis, and dual-threshold strategies is employed to conduct in-depth analysis of defect quantity, size, and type, generating comprehensive batch inspection reports. By implementing object detection technology in polymer film manufacturing environments, this study significantly enhances defect detection accuracy, providing robust support for industrial quality control. The proposed solution not only resolves multiple limitations of traditional detection methods but also delivers more reliable quality assurance for market-ready products. Specifically, we employ the YOLO (You Only Look Once) (Redmon et al., 2016) architecture as the foundational network. The main contributions of this paper are as follows:

1. We embedded the CBAM attention mechanism into high-level networks of YOLOv8. CBAM simultaneously filters features from both channel and spatial dimensions, enabling comprehensive and refined capture of critical information, thereby enhancing feature extraction and detection accuracy for small defects.
2. We adopted the Mean Teacher semi-supervised learning method to address data labeling challenges, effectively mining latent features from limited labeled data and abundant unlabeled data to achieve high precision with small datasets.
3. Recognizing the limitations of traditional IoU loss in capturing scale variations of micron-scale defects, we implement a normalized Wasserstein distance (NWD) (Wang et al., 2021a)-based loss function to optimize neural network training. This modification enables effective handling of complex scenarios and improves small target recognition.
4. We propose a multi-threshold mask-segmentation algorithm to detect and delineate the areas covered by mask-segmentation values in defective images. By exploiting pixel connectivity to distinguish foreground from background, the algorithm accurately extracts defect contours.

## 2 Related work

Machine vision-based defect detection methods primarily rely on cameras, lighting systems, and image processing algorithms. Common defect detection approaches include edge detection, texture analysis, defect region segmentation, and deep learning, which analyze and process images to identify surface or structural defects. Rawashedeh et al. (2023) develops vision-based quality metrics for detecting the defects of width consistency, film edge straightness, and specks in a polymeric film production process. Nakashima et al. (2021) introduce a CNN design tool to detect defects that appear in the manufacturing process of wrap film products. While vision-based defect detection methods have been extensively explored using various image processing algorithms, significant challenges remain in micron-scale defect recognition and small-sample learning with limited labeled data.

### 2.1 Deep learning in defect detection

Object detection methods can generally be categorized into two main types. The first is region-based two-stage detection models, such as region-based Fast R-CNN (Girshick, 2015), Region-based Fully Convolutional Network (R-FCN) (Dai et al., 2016), and Mask R-CNN (He et al., 2017). The second category comprises regression-based one-stage detection methods, exemplified by the YOLO (You Only Look Once) series, Single Shot MultiBox Detector (SSD) (Liu et al., 2016), and RetinaNet (Lin et al., 2017). One-stage methods directly predict object categories and estimate bounding boxes without requiring preliminary region proposals, thereby reducing intermediate steps compared to two-stage approaches.

Researchers worldwide have dedicated efforts to defect detection across various devices and material surfaces, focusing on defect regions, sizes, quantities, and types. For instance, Kou et al. (2021) proposed a CNN-based method for detecting defects in LCD screens of electric meters, combining character defect detection with OCR recognition. Chen S. et al. (2021) introduced a transfer learning-based method for efficient wafer map defect recognition. Cao et al. (2022) proposed an improved Cascade R-CNN model to address challenges in detecting small-scale, low-contrast tile defects under diverse texture backgrounds. Sun et al. (2024) integrated an analytic hierarchy process (AHP)-based model to quantify pit defect features. Das and Deka (2023) developed “Seg-YOLO” to combine defect detection with pixel-level segmentation for handloom fabrics. Li et al. (2023) designed a Global Channel and Spatial Context (GCSC) module to enhance detection of subtle defects through self-attention mechanisms. Xu et al. (2024) proposed a surface defect detection model in filled vials named ESMNet based on YOLOv7-tiny by integrating the ELAN-SC module and the Multi-Scale Cross Fusion Attention (MCF) module , which achieves a 0.8% improvement in mean Average Precision (mAP) over state-of-the-art methods while maintaining low computational complexity. Hou et al. (2024) developed a DCNN-based approach for solar cell fault detection, utilizing a network architecture with three convolutional layers, one pooling layer, one fully connected layer, and an output layer. Laidi and Bouanani (2024) created an automated tool for detecting and correcting pixel-level anomalies in raw satellite imagery. Xu et al. (2025a) proposed CSLNet based on YOLOv8n by integrating the C2f_Starmodule and the Lightweight Fusion Module(LFM) to detect foreign objects in lyophilized powder, enabling adaptive multi-scale feature aggregation with cross-level correlation. Xu et al. (2025b) propose Defect Detection of Surface and Contents in Vials(DDSCNet) by designing Quadra Fusion and Attention (QUFUAtt) module which enhances the capability of feature fusion in network, introducing the self-attention and convolution (ACmix) which focuses on the defective areas, and Linear Deformable Convolution which extracts the weak features of defects. Numerous researchers have leveraged deep learning techniques to propel the advancement of defect detection.

### 2.2 Tiny object detection

Tiny object detection is inherently more challenging than generic object detection, prompting extensive research aimed at enhancing recognition performance. Gong et al. (2021) propose a novel concept, fusion factor, to control information that deep layers deliver to shallow layers,for adapting FPN to tiny object detection. Wang et al. (2021b) propose a multiple center points based learning network (M-CenterNet) to improve the localization performance of tiny object detection in aerial images. Xiao et al. (2023) proposed a novel feature pyramid composite neural network structure comprising the context enhancement module (CEM) and feature purification module (FPM) to detect tiny object. Guo et al. (2023) propose a Hierarchical Activation (HA) method to obtain scale-specific feature subspaces by activating object features at different scales hierarchically. In small target detection, since the target usually occupies a smaller number of pixels in the image, traditional Intersection over Union (IoU) (Girshick et al., 2014; Zhang et al., 2023) loss might not succeed in effectively distinguish similar small targets, which can cause a reduction in the detection accuracy. The Wasserstein distance, on the other hand, by capturing the distribution of small target features, can more accurately evaluate the variation between the predicted result and the true frame and thus optimize the model to perform better in identifying small targets (Cai et al., 2023).

### 2.3 Semi-supervised learning

The sparse and limited features of small targets make them difficult to extract using existing methods, while the complex production processes of polymer films complicate data collection and annotation. Zheng et al. (2022) introduced a new semi-supervised learning framework, SimMatch, which simultaneously considers semantic similarity and instance similarity. Zhang et al. (2021) propose Curriculum Pseudo Labeling (CPL), a curriculum learning approach to leverage unlabeled data according to the model's learning status. They apply CPL to FixMatch and call this improved algorithm FlexMatch. Wang et al. (2022) propose FreeMatch to adjust the confidence threshold in a self-adaptive manner according to the model's learning status, which boost the performance of imbalanced SSL. Jiang et al. (2022) propose a novel learning framework, called Multiple Graph Learning Neural Networks (MGLNN), for multiple graph learning and multi-view semi-supervised classification. Xu et al. (2021) develop a simple yet powerful framework Dash, whose key idea is to select a subset of training examples from the unlabeled data when performing existing SSL methods so that only the unlabeled examples with pseudo labels related to the labeled data will be used to train models. Studies on semi-supervised learning have enabled fully effective training with only sparsely annotated data.

## 3 Materials and methods

### 3.1 Problem statement

During the production of polymer material films, factors such as manufacturing processes, raw material quality, and environmental conditions can lead to micron-scale surface defects like crystalline spots and black dots. These defects pose significant challenges for traditional detection methods in terms of effective identification and classification. Current machine learning-based detection approaches widely used in industrial production face the following limitations:

- Insufficient Detection Accuracy: Traditional machine learning methods rely on manual feature extraction, resulting in poor recognition performance for complex defect morphologies and lighting variations, with frequent false positives and missed detections.
- Limited Generalization Capability: Existing algorithms struggle to adapt to defect variations across production batches and environmental conditions, requiring frequent parameter adjustments and incurring high maintenance costs.

To address these issues, this study introduces a deep learning-based automated defect detection framework. Compared to traditional approaches, deep learning models autonomously learn multi-scale defect features, thereby enhancing detection accuracy and generalization. However, small target detection remains challenging for polymer film inspection tasks. Due to the minute size of defects, standard object detection algorithms often suffer from information loss when processing small targets, leading to suboptimal performance. Accordingly, we propose an enhanced YOLOv8 architecture tailored for small object detection scenarios, with modifications focused on improving detection accuracy.

### 3.2 Network architecture

**Figure 1:**
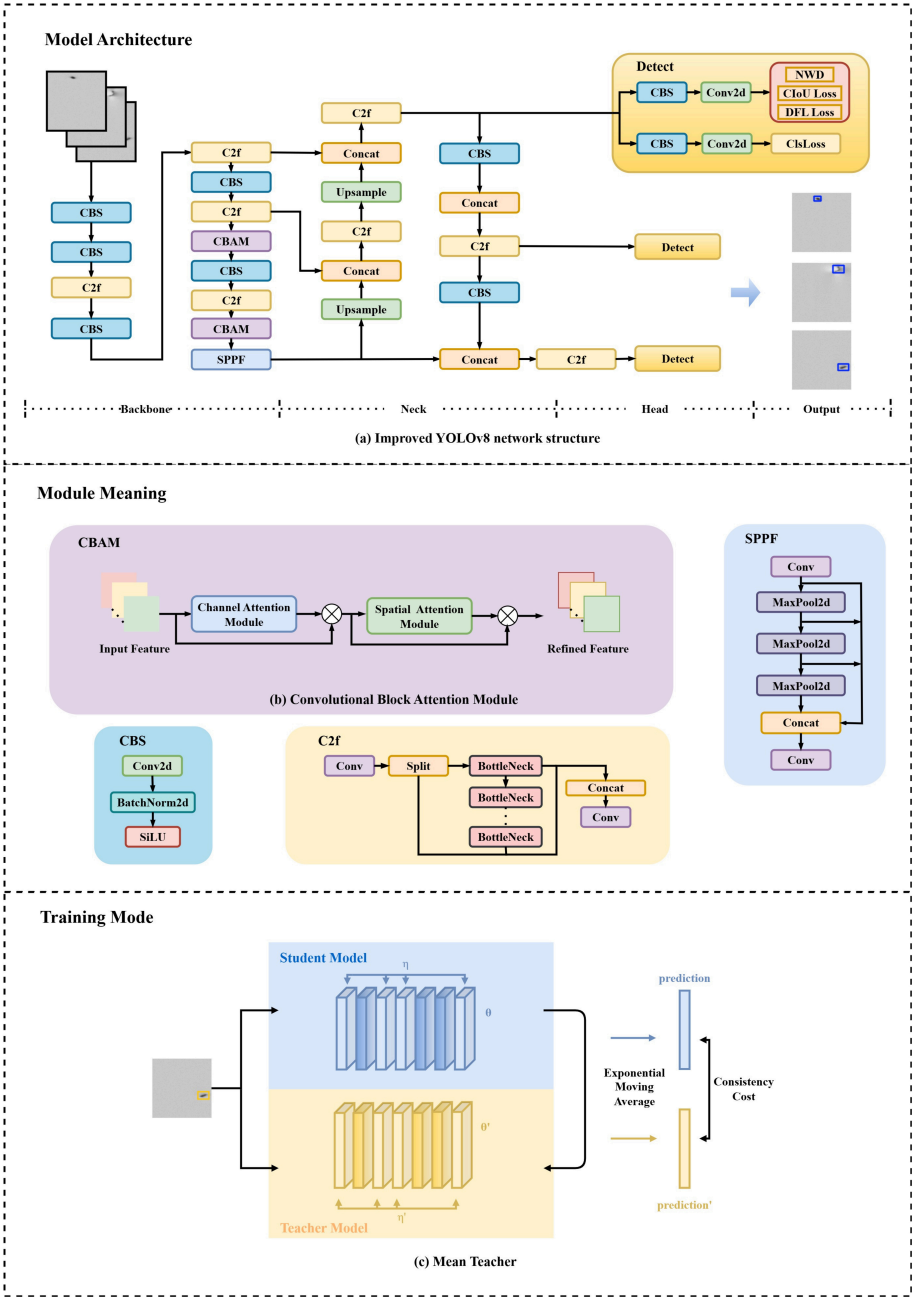
Target detection model framework for defect detection. **(a)** The network architecture employs YOLOv8 as the backbone, incorporates the CBAM module in high-level networks, and combines the Normalized Wasserstein Distance loss function. **(b)** The CBAM module comprises both channel attention and spatial attention mechanisms. **(c)** The method employs MeanTeacher for semi-supervised training, utilizing YOLOv8 as the student model. The teacher model parameters are updated via Exponential Moving Average.

In industrial inspection, detection accuracy is paramount, and the YOLO algorithm has become the preferred foundational framework due to its unique advantages. Its single-stage detection architecture predicts target categories and locations in a single forward pass, employing an end-to-end learning approach that eliminates complex feature engineering, enabling the model to directly map images to detection results. Trained on large-scale datasets, YOLO exhibits strong generalization across diverse targets, with successive versions continuously improving performance. This study adopts YOLOv8 as the base architecture and proposes a YOLO-based object detection method incorporating Normalized Wasserstein Distance (NWD), as illustrated in Figure 1. The YOLOv8 architecture comprises three components: a Backbone, Neck, and Head for feature extraction, fusion, and final prediction, respectively. Processed images enter the Backbone, which utilizes CBS (Convolution-BatchNorm-SiLU), C2f, and SPPF (Spatial Pyramid Pooling Fast) modules based on an enhanced CSPDarknet. The CBS module, consisting of a convolutional layer (Conv2D), batch normalization (BatchNorm2D), and SiLU activation, focuses on local feature extraction. In the C2f module, input features undergo multi-level splitting, where partial features are directly propagated while others pass through multiple Bottleneck operations before fusion, enhancing feature reuse efficiency. The SPPF module employs multiple MaxPool layers for spatial pyramid pooling (SPP) to capture multi-scale features. The Neck integrates a Feature Pyramid Network (FPN) and Path Aggregation Network (PAN) to strengthen multi-scale detection capabilities. The Head performs final classification and bounding box regression, outputting target category probability distributions, predicted bounding box coordinates, and confidence scores. As shown in Figure 1, this study optimizes the model by combining NWD with classification loss (ClsLoss), Distribution Focal Loss (DFL), and Complete Intersection over Union Loss (CIoU), while embedding the CBAM (Convolutional Block Attention Module) attention mechanism to achieve higher precision in small target detection and complex scenarios. In real-world scenario testing, the model achieves real-time processing of 16K resolution images on an NVIDIA 3090 GPU. Specifically, the measured latency is approximately 1.4 milliseconds (ms) with a throughput of 714 frames per second (FPS).

### 3.3 Convolutional block attention module

**Figure 2:**
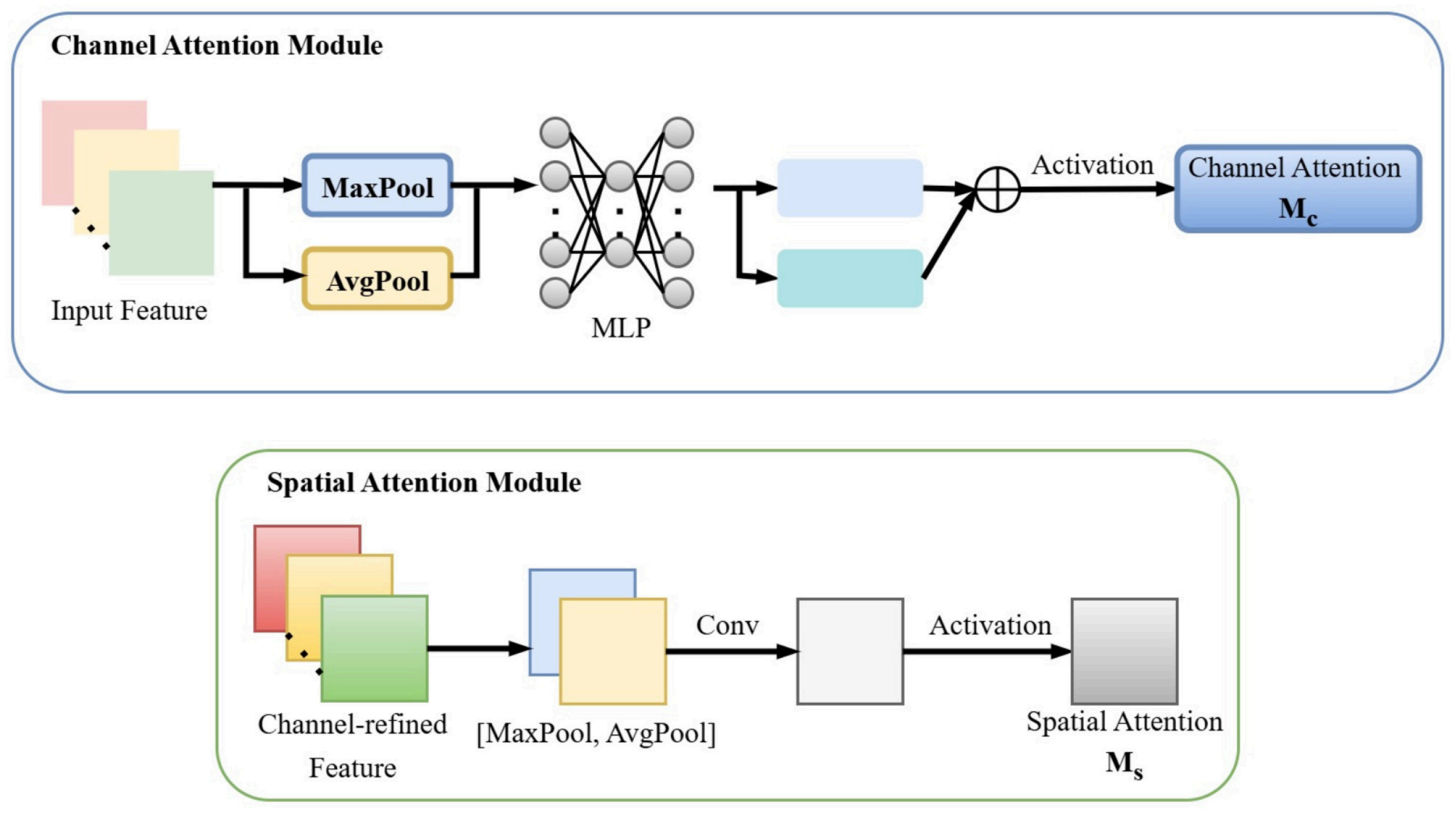
Channel attention and Spatial Attention in CBAM attention mechanism.

The CBAM (Convolutional Block Attention Module) comprises a Channel Attention (CA) mechanism and a Spatial Attention (SA) mechanism, as illustrated in Figure 2. These components enhance the model's perception capabilities by adaptively adjusting feature map responses at both global (channel-wise) and local (spatial) levels. By computing channel and spatial attention weights, CBAM strengthens the expressive power of target regions, thereby improving detection accuracy.

Given an intermediate feature map **F**∈ℝ^*C*×*H*×*W*^ as input, CBAM module infers a 1D channel attention map ${\text{M}}_{c}\in {\mathbb{R}}^{C\times 1\times 1}$ and a 2D spatial attention map ${\text{M}}_{s}\in {\mathbb{R}}^{1\times H\times W}$.


(1) $$F^{\prime }=M_{c}(F)⊗F$$

(2) $$F^{\prime \prime }=M_{s}(F^{\prime })⊗F^{\prime }$$

Here, ⊗ denotes element-wise multiplication, and ***F*****″** represents the final optimized output.

The Channel Attention Module (CAM) aggregates spatial information from feature maps using both average pooling and max pooling, generating two distinct spatial context representations: ${\text{F}}_{\text{avg}}^{c}$ and ${\text{F}}_{\text{max}}^{c}$, corresponding to average-pooled and max-pooled features. These features are fed into a shared network layer composed of a multi-layer perceptron (MLP) with one hidden layer to produce the channel attention map ${\text{M}}_{c}\in {\mathbb{R}}^{C/r\times 1\times 1}$. The hidden activation size is set to ℝ^*C*/*r*×1 × 1^, where *r* is the reduction ratio. The channel attention is computed as:


(3) $$\begin{array}{l} {\text{M}}_{c}\left(\text{F}\right)=\sigma \left(\text{MLP}\left(\text{AvgPool}\left(\text{F}\right)\right)+\text{MLP}\left(\text{MaxPool}\left(\text{F}\right)\right)\right) \end{array}$$

where σ denotes the sigmoid function.

The Spatial Attention Module (SAM) aggregates channel-wise information by applying average pooling and max pooling across the channel dimension, generating two 2D feature maps: ${\text{F}}_{\text{avg}}^{s}\in {\mathbb{R}}^{1\times H\times W}$ and ${\text{F}}_{\text{max}}^{s}\in {\mathbb{R}}^{1\times H\times W}$, which represent the channel-wise average-pooled and max-pooled features, respectively. These maps are concatenated and processed via a standard convolutional layer to produce the 2D spatial attention map. The spatial attention is formulated as:


(4) $$\begin{array}{l} {\text{M}}_{s}\left(\text{F}\right)=\sigma \left(f^{7\times 7}\left(\left[\text{AvgPool}\left(\text{F}\right);\text{MaxPool}\left(\text{F}\right)\right]\right)\right) \end{array}$$

where *f*^7 × 7^ denotes a convolutional operation with a 7 × 7 filter.

In this study, the CBAM module is integrated into the high-level network structures of YOLOv8's backbone. This enhancement strengthens the model's focus on small targets in high-level feature maps by incorporating fine-grained details from lower-level layers, thereby improving detection performance. The Channel Attention Module extracts global statistical features from each channel through global max-pooling and average-pooling operations, and employs a shared Multi-Layer Perceptron to generate a channel-wise weight vector. This vector quantifies the importance of different channels, assigning high weights to channels that exhibit salient defect-related features while suppressing those dominated by background interference. The Spatial Attention Module aggregates max-pooled and average-pooled features along the channel dimension to produce a two-dimensional spatial weight map. This map quantifies the significance of each spatial location in the image, enabling the network to focus on local high-response pixels within the defect regions.

### 3.4 Mean teacher

In object detection tasks, acquiring labeled data is costly, particularly in industrial inspection domains such as defect detection in polymer material films. Each annotated sample contains geometric parameters of the bounding box, represented in the center-coordinates format (center-x, center-y, width, height). This parameterization method follows the standard annotation protocol for object detection tasks in computer vision, as adopted by major datasets (e.g., COCO, Pascal VOC). To effectively utilize limited labeled data and fully exploit the latent information in unlabeled data, this study adopts the *Mean Teacher* method to enhance the generalization capability of the detection model.

The Mean Teacher framework is a semi-supervised learning approach based on a *teacher-student architecture*. This framework uses a *Mean Teacher* approach to leverage both labeled and unlabeled data, improving generalization by enforcing prediction consistency between a student and a slowly-updated teacher model. The *student model* is trained on labeled data via standard supervised learning, while consistency loss constraints are applied to its predictions on unlabeled data, as illustrated in Figure 1. The consistency loss *J* is defined as the expected distance between the predictions of the student model (with weights θ and noise η) and those of the teacher model (with weights θ′ and noise η′):


(5) $$\begin{array}{l} J\left(\theta \right)={\text{E}}_{\text{x},\eta^{\prime },\eta }\left[||f\left(\text{x},\theta^{\prime },\eta^{\prime }\right)-f\left(\text{x},\theta ,\eta \right)|{|}^{2}\right] \end{array}$$

The teacher model updates its weights using *Exponential Moving Average (EMA)* to ensure parameter stability and guide the student model's learning. At training step *t*, the teacher weights $\theta_{t}^{\prime }$ are updated as the EMA of the student weights θ, with α as the smoothing hyperparameter:


(6) $$\begin{array}{l} \theta_{t}^{\prime }=\alpha \theta_{t-1}^{\prime }+\left(1-\alpha \right)\theta_{t} \end{array}$$

Compared to fully supervised training, the Mean Teacher method reduces reliance on extensive manual labeling in semi-supervised object detection tasks, achieving robust performance even under high annotation costs. For this study, YOLOv8 serves as the student model, with the teacher model weights updated asynchronously via EMA. During the training During the training process, the dataset is partitioned into labeled and 0.5:0.5. The student model computes classification loss, localization loss, and confidence loss based on labeled data, while aligning its predictions with the teacher model's outputs on unlabeled data through consistency loss. This approach optimizes the use of limited labeled samples while leveraging unlabeled data to improve detection robustness and adaptability.

### 3.5 Object detection method with the normalized Wasserstein distance algorithm

For the tiny object detection problem, Wasserstein distance is an effective metric for measuring the differences between two distributions, which is particularly suitable for dealing with small targets. Compared to IoU loss, Wasserstein distance relies less on the exact overlap of boundaries and focuses on the overall distribution of features, which makes the model more robust to small target detection. In addition, Wasserstein distance provides a smooth loss curve, which makes the model optimization process more smooth and stable. This is especially important for the case of small targets with fewer samples because, in a sample-scarce environment, the training process tends to be unstable, and the Wasserstein distance can effectively alleviate this problem, thus improving the convergence speed of training and the model's ultimate performance. Through this approach, the target detection model can better adapt to the characteristics of small object, optimize recognition performance, and finally realize the efficient recognition of small targets. This not only boosts the model's performance to be applied in complex scenarios, but also provides a more reliable solution for practical industrial applications.

In target detection, the NWD can be used to evaluate the difference between the predicted box and the ground truth box. Compared with the widely used IoU loss (Han et al., 2017; Dai et al., 2025), the NWD is more sensitive to the full range of differences in box position, shape, size, etc., and it does not suffer from the problem of zero gradient, which provides meaningful gradient for optimization even if there is no intersection between the predicted and ground truth boxes.

For Gaussian distributions, where Na and Nb are formulated by the bounding boxes *A* = (*cx*_*a*_, *cy*_*a*_, *w*_*a*_, *h*_*a*_) and *B* = (*cx*_*b*_, *cy*_*b*_, *w*_*b*_, *h*_*b*_), the previous equation simplifies to


(7) $$\begin{array}{l} W_{2}^{2}\left(N_{a},N_{b}\right)=\|\left({\left[\begin{array}{cccc} cx_{a}, & cy_{a}, & \frac{w_{a}}{2}, & \frac{h_{a}}{2} \end{array}\right]}^{\text{T}},{\left[\begin{array}{cccc} cx_{b}, & cy_{b}, & \frac{w_{b}}{2}, & \frac{h_{b}}{2} \end{array}\right]}^{\text{T}}\right){\|}_{2}^{2} \end{array}$$

The NWD is a normalized measure of the Wasserstein distance in exponential form, as a way to decrease the model's dependence on position and size.


(8) $$\begin{array}{l} NWD\left(N_{a},N_{b}\right)=\exp\left(-\frac{\sqrt{W_{2}^{2}\left(N_{a},N_{b}\right)}}{C}\right) \end{array}$$

The NWD metric form is formulated as the loss function of the target detection box, *N*_*p*_ denotes the Gaussian distribution model of the predicted box *p*, and *N*_*g*_ denotes the Gaussian distribution model of the ground truth box *G*. The loss function is as follows:


(9) $$\begin{array}{l} {\text{L}}_{NWD}=1-NWD\left(N_{p},N_{g}\right) \end{array}$$

In this work, we use a target detection model incorporating the NWD loss function as a gradient optimization method to provide greater robustness to small target detection. During training, we use a bounding box loss function incorporating the NWD loss to compute the loss and use the other losses of the target detection model in combination, as illustrated in Figure 1, to optimize the behavior of the whole model. We evaluate the effectiveness of the NWD loss on the performance of small-scale object detection through several rounds of experiments and compare it with the traditional IoU loss.

NWD loss can better capture scale and shape changes in the target frame. The traditional IoU or L2 loss is difficult to accurately reflect the differences in frames when the target frame size or aspect ratio changes significantly, while NWD can handle these situations more robustly by taking into account the overall displacements and scale changes between frames. Especially in scenarios where the target objects are unevenly sized or drastically changing, NWD enables the model to adapt more effectively to detecting targets at different scales. When the predicted box and the ground-truth box do not overlap or the object is extremely tiny, let *B*_*p*_ denotes the predicted bounding box and *B*_*g*_ denotes the ground-truth bounding box, $\text{IoU}=\frac{\left|B_{p}\cap B_{g}\right|}{\left|B_{p}\cup B_{g}\right|}=0$, the gradient of the IoU loss is identically zero, halting further optimization. In contrast, regardless of overlap, the Wasserstein distance *W* is always positive, as $W_{2}^{2}\left(N_{p},N_{g}\right)>0$. So the NWD loss continues to provide a non-zero gradient that ensures sustained convergence throughout training. We propose a hybrid bounding box loss function that integrates both IoU and NWD metrics. This loss function dynamically balances the contributions of both metrics through a learnable parameter α, with the complete computation procedure formalized in Algorithm 1.

**Algorithm 1:**
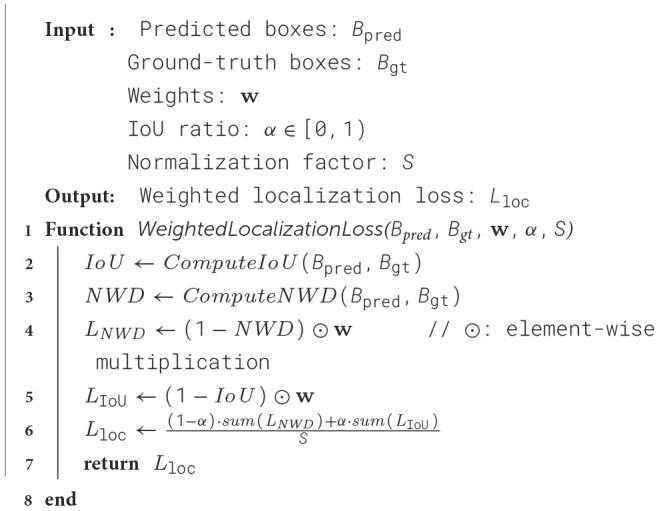
Weighted Localization Loss with NWD and IoU.

### 3.6 Multi-threshold mask segmentation algorithm

The real-time demand for image processing in large-scale industrial production is very high, and the detection algorithm designed in this study uses a multi-threshold mask segmentation strategy to detect and segment the region covered by the mask segmentation value in an image. It is not only able to detect pixels that conform to a certain range, but also can be extended to pixels that conform to other ranges. This multi-threshold processing improves the robustness of the method, improves the system's ability to recognize defects of different types or intensities, and also flexibly adjusts the upper and lower limit values according to the different detection needs, adapting to the detection tasks in a variety of different scenarios.

In practical detection, the input image *I*(*x, y*) is defined as a grayscale image, the upper threshold is *U*_*threshold*_, the lower threshold is *L*_*threshold*_, and the boundaries of the middle two masks define the thresholds as *M*_*threshold*1_, *M*_*threshold*2_. generating the masks *M*_1_, *M*_2_ as:


(10) $$\begin{array}{l} M_{1}(x,y)=\{\begin{array}{l} 1,\;L_{\text{threshold}}\leq I(x,y)\leq M_{\text{threshold1}}\\ 0,\text{ otherwise } \end{array}\\ M_{2}(x,y)=\{\begin{array}{l} 1,\;M_{\text{threshold2}}\leq I(x,y)\leq U_{\text{threshold}}\\ 0,\text{ otherwise} \end{array} \end{array}$$

The merge mask is *M*:


(11) $$\begin{array}{l} M\left(x,y\right)=M_{1}\left(x,y\right)\vee M_{2}\left(x,y\right) \end{array}$$

Based on the connectivity principle, our algorithm determines which pixels belong to the boundary N of the same object by analyzing the neighborhood relationship of pixels in the image. It exploits the connectivity of the pixels to identify the differences between the foreground (the object) and the background in order to extract the contours of the defects, the procedure is formalized in Algorithm 2.


(12) $$\begin{array}{c} N(x,y)=\{(x^{\prime },y^{\prime })|\max\{(|M(x^{\prime },y)-M(x,y)|,|M(x,y^{\prime })-M(x,y)|)\}\\ =1\} \end{array}$$

**Algorithm 2:**
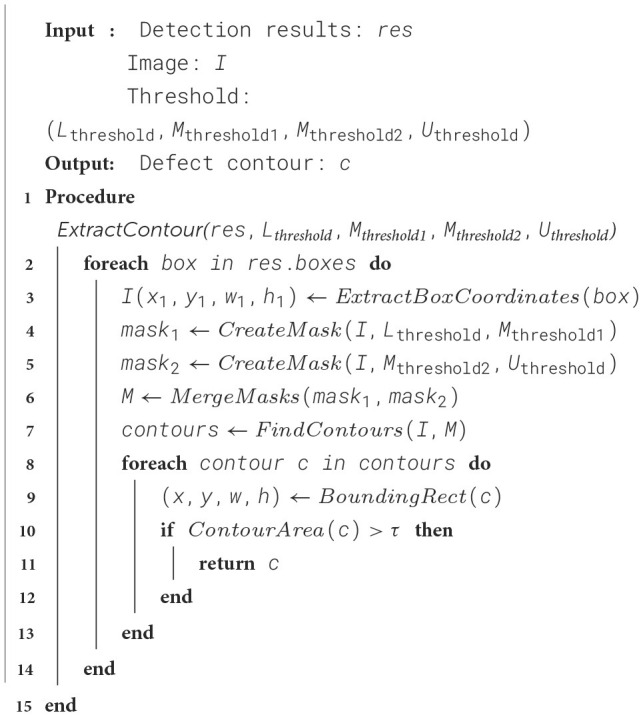
Multi-threshold Mask Segmentation Algorithm.

**Figure 3:**
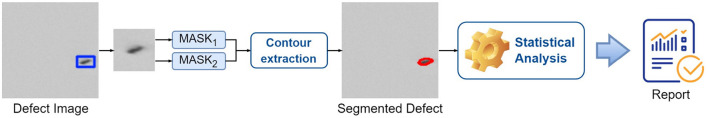
Multi threshold mask segmentation workflow.

In Figure 3, our defect detection system automatically identifies defects through real-time image acquisition and applies advanced deep learning algorithms. By utilizing a multi-threshold mask segmentation strategy to extract contours, the system further quantifies the number and size of defects to analyze the distribution patterns and area proportions of different defect types. This approach not only enhances detection sensitivity and accuracy but also improves the classification capability for diverse defects in complex scenarios. Through setting multiple thresholds to separately detect dark and bright defects, the system further categorizes them into minor, moderate, and severe defects. After detailed statistical analysis of defect types and quantities, a statistical model is constructed based on the detected defect counts and their spatial distribution within the image. Additionally, thresholds are dynamically adjusted per production batch using historical defect size distributions. This accommodates material process variations.

## 4 Results

### 4.1 Datasets description

**Figure 4:**
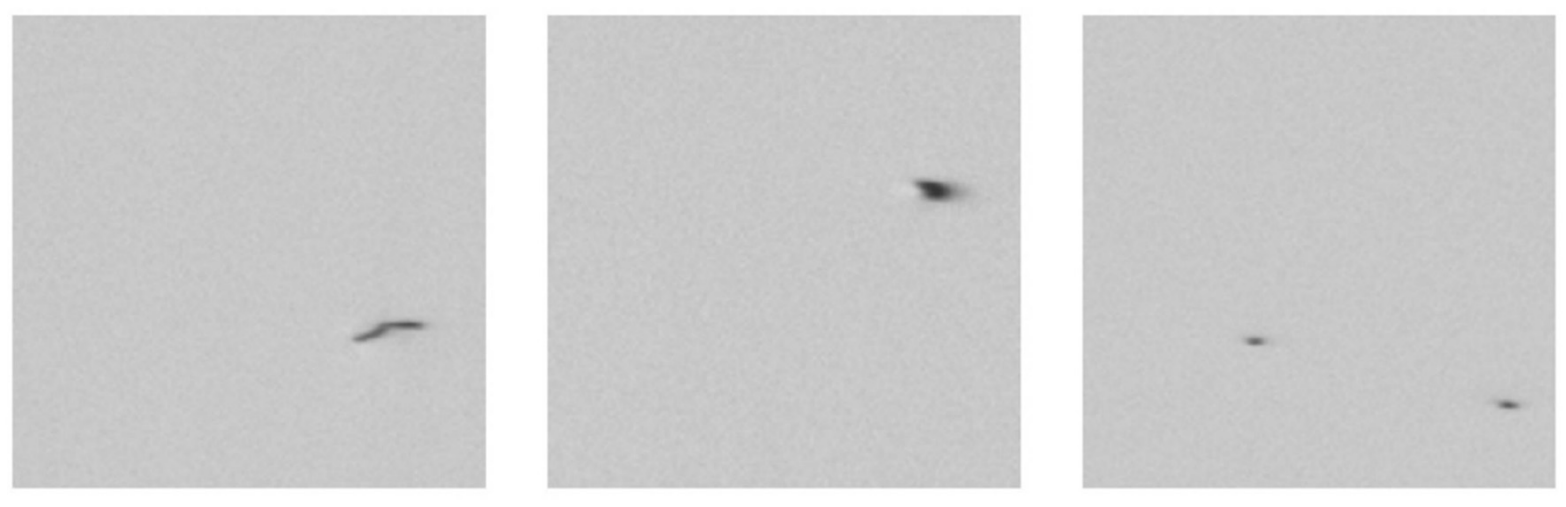
Typical examples of polymer thin film defects.

The polymer material film studied here is colorless and transparent. The primary detection targets are defects on the film surface, appearing as irregular black or dark gray microspots (“crystalline points”). Defect diameters range from 0 to 800 microns, occupying only a few pixels in high-resolution images. The dataset comprises 8,883 images, divided into a training set of 6,218 images (4,701 defective and 1,517 defect-free) and a test set of 2,665 images (1,917 defective and 748 defect-free). Typical defect samples are illustrated in Figure 4.

To enhance dataset diversity and alleviate data imbalance, this study employs an adaptive blur and brightness adjustment method for data augmentation. This approach applies varying degrees of blur and brightness transformations to random regions of images to simulate complex environmental variations, thereby improving the model's generalization capabilities. The method randomly selects a blur intensity value and ensures that the convolution kernel size is an odd integer to guarantee the effectiveness of Gaussian blur. By reducing local details through Gaussian blur, it simulates scenarios such as blurred defects, lighting changes, or motion blur. For brightness adjustment, a rectangular region is randomly selected on the image, and different brightness scaling factors are applied to modify the grayscale values of the region, mimicking uneven illumination, localized reflections, or exposure variations. Through this method, data augmentation is achieved without requiring additional data collection, enabling the model to better adapt to complex variations in real-world production environments and improving the reliability and stability of defect detection.

### 4.2 Experimental platform

Data annotation environment:

- Operating System: Windows 11 (Intel(R) Core(TM) i5-8265U CPU @ 1.60GHz-1.80GHz)
- Memory: 8GB RAM
- GPU: NVIDIA GeForce MX 150 (2GB VRAM)
- Software: Jinglingbiaozhu (http://www.jinglingbiaozhu.com/, Version2.0.4)

Experimental Training and Validation Environment:

- Operating System: Ubuntu 22.04.4 (Intel(R) Xeon(R) Gold 5318Y CPU @ 2.10GHz)
- Software Stack: CUDA 12.2, cuDNN 8.9, PyTorch 2.4.0
- GPU: NVIDIA GeForce RTX 3090 (24GB VRAM)

### 4.3 Experimental parameters

**Figure 5:**
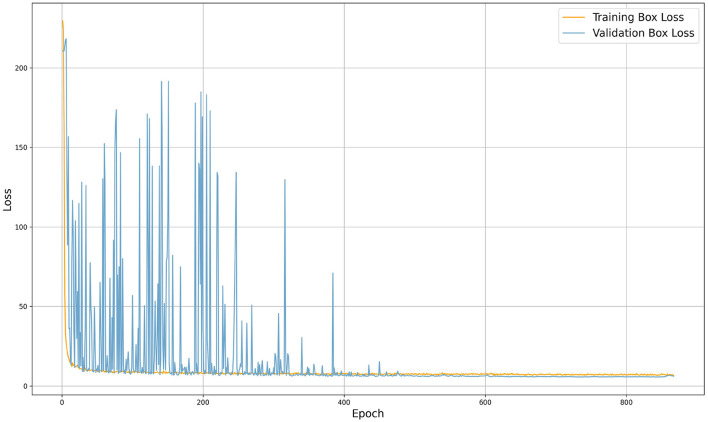
Box Loss convergence over epochs during training and validation.

After acquiring 16K-resolution images, precise annotations were performed using Jingling annotation software, with labeled data converted into TXT files in center-point coordinate format for network training. Throughout the training process, this research utilize a piecewise learning rate schedule incorporating warm-up and annealing, and the training essentially converged after the 866th epoch, as illustrated in Figure 5. Multiple adjustable hyperparameters (e.g., learning rate, batch size, optimizer settings) provide flexibility and adaptability for optimizing model performance, as detailed in Table 1.

**Table 1:** Hyperparameters for model training.

**Hyperparameter**	**Value**
Batch size	512
Image size	128
Warmup initial learning rate	0.01
Warmup bias learning rate	0.1
Warmup momentum	0.8
Warmup epochs	3.0
Mosaic Augmentation	1.0

### 4.4 Evaluation metrics

In industrial defect detection, mAP, Precision and Recall are the key evaluation metrics for measuring the performance of target detection algorithms. Precision is calculated using the following formula:


(13) $$\begin{array}{l} Precision=\frac{TP}{TP+FP} \end{array}$$

where TP represents the number of defects accurately detected by the model, and FP represents the number of defects incorrectly identified. A high Precision score suggests that the majority of the defects detected by the model are actual defects, which effectively reduces false alarms. Excessive false alarms not only increase the production cost, but also prolong the overhaul time of the equipment.

Recall serves as a key evaluation metric, reflecting the ratio of true defects accurately identified by the model. AP is obtained from the Precision-Recall curve. By changing the confidence threshold, different Precision and Recall rates are obtained. The formula is as follows:


(14) $$\begin{array}{l} Recall=\frac{TP}{TP+FN}, \end{array}$$

where FN represents the number of actual defects missed by the model.


(15) $$\begin{array}{l} AP=\int_{0}^{1}p\left(r\right)dr, \end{array}$$

where p refers to precision, and r refers to recall. mAP calculates the Average Precision (AP) under multiple thresholds derived from the Precision and Recall curves and then averages them out. It reflects the detection accuracy of the model under various confidence thresholds by integrating the value of the model under multiple thresholds. For industrial defect detection, a high mAP means that the model can stably detect defects under different conditions and adapt to different detection scenarios. mAP is calculated as follows:


(16) $$\begin{array}{l} mAP=\frac{\overset{n}{\underset{i=1}{\sum }}AP_{i}}{n}, \end{array}$$

### 4.5 Ablation experiment

In the ablation study, we primarily employed mAP@0.5, Precision, and Recall as metrics to evaluate model performance. We selected three baseline models from the YOLOv5 series: YOLOv5n, YOLOv5m, and YOLOv5l. As shown in Table 2, with the incorporation of the NWD (Normalized Wasserstein Distance) loss, the improved models—YOLOv5n-NWD, YOLOv5m-NWD, and YOLOv5l-NWD—achieved 4.62%, 4.0%, and 6.08% increases in mAP@0.5 compared to their baseline counterparts, demonstrating superior accuracy. Notably, Recall values improved by 11.12%, 6.66%, and 11.07% for the three models, respectively. This enhancement is attributed to the NWD method's ability to reduce false negatives, thereby improving defect detection sensitivity.

**Table 2:** Performance comparison of normalized Wasserstein distance algorithm in YOLOv5 series models.

**Networks**	**Param. (M)**	**FPS (f·s^−1^)**	**mAP@0.5**	**Precision (%)**	**Recall (%)**
YOLOv5n	2.6	148	66.63	78.86	49.69
YOLOv5m	25.1	118	68.54	78.46	54.66
YOLOv5l	53.2	89	67.65	77.32	51.69
YOLOv5n-NWD	2.6	149	71.25	72.16	60.81
YOLOv5m-NWD	25.1	118	72.54	75.81	61.32
YOLOv5l-NWD	53.2	89	73.73	75.90	62.76

Among the YOLOv8 series models, the NWD (Normalized Wasserstein Distance) demonstrates superior capability in detecting small-target defects, achieving higher mAP@0.5 values. We conducted similar ablation experiments on the YOLOv8 series. As shown in Table 3, the improved models—YOLOv8n-NWD, YOLOv8m-NWD, and YOLOv8l-NWD—exhibit 7.07%, 7.69%, and 2.95% increases in mAP@0.5 compared to their baseline counterparts when trained with the NWD loss. These improvements are accompanied by enhancements in both Precision and Recall. Specifically, YOLOv8l-NWD achieves an 8.61% increase in Precision over YOLOv8l, while YOLOv8m-NWD improves Recall by 8.62% compared to YOLOv8m, highlighting the balanced performance gains enabled by the NWD framework.

**Table 3:** Performance comparison of normalized Wasserstein distance algorithm in YOLOv8 series models.

**Networks**	**Param. (M)**	**FPS (f·s^−1^)**	**mAP@0.5**	**Precision (%)**	**Recall (%)**
YOLOv8n	3.4	136	79.61	85.52	79.61
YOLOv8m	25.5	97	81.13	87.51	81.13
YOLOv8l	43.7	64	84.94	78.43	84.94
YOLOv8n-NWD	3.4	139	86.68	86.48	81.28
YOLOv8m-NWD	25.5	97	88.82	78.85	89.75
YOLOv8l-NWD	43.7	64	87.89	87.04	83.24

Regarding the role of different loss functions in this study, we conducted an experiment of the application of various loss functions on the YOLOv8n model. Table 4 illustrates the impact of different loss functions on model performance, including DIoU, GIoU, and NWD. The data reveal that the model utilizing the NWD loss function performs the best in terms of mAP@0.5, outperforming DIoU by 3.21% and surpassing GIoU by 2.98%.

**Table 4:** Comparison of results using different loss functions on YOLOv8n.

**Networks**	**Param. (M)**	**FPS (f·s^−1^)**	**mAP@0.5**	**Precision (%)**	**Recall (%)**
YOLOv8n	3.4	136	79.61	85.52	79.61
YOLOv8n + DIoU	3.4	138	83.47	84.35	78.40
YOLOv8n + GIoU	3.4	139	83.70	74.20	85.14
YOLOv8n + NWD	3.4	139	86.68	86.48	81.28

Furthermore, we conducted an in-depth exploration of the effects of integrating various attention mechanisms into the YOLOv8n model. Table 5 presents the changes in model performance after incorporating SE (Squeeze-and-Excitation), CA (Coordinate Attention), and CBAM (Convolutional Block Attention Module) attention mechanisms. The data indicates that the model equipped with the CBAM attention mechanism achieved the best performance across all key metrics. Specifically, the mAP@0.5 of CBAM outperformed SE by 4.51% and outperformed CA by 2.6%.

**Table 5:** Comparison of results with different attention mechanisms.

**Networks**	**Param. (M)**	**FPS (f·s^−1^)**	**mAP@0.5**	**Precision (%)**	**Recall (%)**
YOLOv8n	3.4	136	79.61	85.52	79.61
YOLOv8n + SE	3.1	133	83.08	66.95	84.48
YOLOv8n + CA	3.2	126	84.99	82.08	80.84
YOLOv8n + CBAM	3.2	129	87.59	85.25	85.25

Table 6 summarizes the ablation studies conducted on YOLOv8n to comprehensively evaluate the incremental improvements introduced into the YOLOv8 model. Specifically, these enhancements include the integration of the NWD loss function, CBAM attention mechanism, and Mean Teacher semi-supervised training method.

**Table 6:** Comparison of ablation experiment results on YOLOv8n.

**YOLOv8n**	**CBAM**	**Mean Teacher**	**NWD**	**Param. (M)**	**FPS (f·s^−1^)**	**mAP@0.5**	**Precision (%)**	**Recall (%)**
✓				3.4	136	79.61	85.52	79.61
✓	✓			3.2	129	87.59	85.25	85.25
✓		✓		3.4	138	83.33	73.40	83.40
✓			✓	3.4	139	86.68	86.48	81.28
✓	✓	✓	✓	3.2	130	87.87	89.38	80.21

The application of the CBAM attention mechanism to the YOLOv8n framework increased the mean Average Precision (mAP) by 7.98% compared to the baseline YOLOv8 model, demonstrating its efficacy in enhancing the detection of subtle and complex features. Incorporating the Mean Teacher semi-supervised training method improved mAP@0.5 by 3.72% over the baseline, highlighting its ability to effectively leverage unlabeled data. Combining NWD, CBAM, and Mean Teacher in the YOLOv8n framework achieved an 8.26% improvement in mAP@0.5 compared to the baseline model. This synergy significantly boosts performance, underscoring the framework's capability for high-precision detection of small targets and extraction of complex defect features.

### 4.6 Comparative experiment

To demonstrate the superiority of YOLOv8-NWD, we compared it against equivalent models from YOLO iterations (YOLOv5, YOLOv6, YOLOv10, YOLOv11, YOLOv12, YOLOv13), RT-DETR (Zhao et al., 2024), SSD and Faster R-CNN (Ren et al., 2016). Table 7 shows that YOLOv8n-NWD achieves higher mAP@0.5 values than all counterparts. The primary improvement lies in Recall, which increased by 31.59%, 30.48%, 30.87%, 28.92%, 31.08% and 26.68%, compared to YOLOv5, YOLOv6, YOLOv10, YOLOv11, YOLOv12 and YOLOv13, respectively. These gains reduce false negatives in industrial settings, ensuring reliable product quality control.

**Table 7:** Performance comparison of different object detection models in industrial defect detection tasks.

**Networks**	**Param. (M)**	**FPS (f·s^−1^)**	**mAP@0.5**	**Precision (%)**	**Recall (%)**
RT-DETR(ResNet101)	61.9	24	34.53	35.41	53.43
Faster R-CNN(VGG16)	137.1	41	30.35	34.46	45.45
Faster R-CNN(ResNet50)	28.4	19	34.39	35.77	50.91
SSD(VGG16)	26.3	198	30.59	52.72	11.60
YOLOv5n	2.6	148	66.63	78.86	49.69
YOLOv6n	4.5	176	67.56	88.09	50.80
YOLOv8n	3.4	136	79.61	85.52	79.61
YOLOv10n	2.7	111	64.72	80.67	50.41
YOLOv11	2.6	122	68.49	86.76	52.36
YOLOv12	2.6	75	66.73	88.13	50.20
YOLOv13	2.5	51	67.73	73.23	53.53
Ours-YOLOv8n	3.2	130	87.87	89.38	80.21

Traditional machine learning methods typically rely on grayscale-based discrimination to identify defects. These approaches analyze pixel intensity differences to separate targets from backgrounds using techniques like threshold segmentation or edge detection, followed by shape analysis (e.g., aspect ratio, contour features) to classify defects (e.g., scratches, bubbles). However, they struggle with lighting variations, noise, and complex backgrounds, leading to high false-positive or false-negative rates. In industrial production, defect detection accuracy is critical for quality assurance. We evaluated detection rates across 11,829 factory-sourced defect images to compare models and traditional methods.


(17) $$\begin{array}{l} \text{Detection rate}=\frac{\text{Number of detected defects}}{\text{Number of total defects}}, \end{array}$$

Table 8 reveals that YOLOv8n-NWD, YOLOv8m-NWD, and YOLOv8l-NWD achieve detection rates exceeding 95% for image sizes of 64 × 64 and 128 × 128 pixels. Notably, the YOLOv8 series outperforms traditional machine learning methods and Deep Q-Network (DQN) (Cao et al., 2022), particularly in detecting small targets. The detection rate for micron-scale defects improves markedly at larger image sizes (e.g., 128 × 128).

**Table 8:** Detection rate performance comparison.

**Method**	**Image size (pixel)**	**Detection rate (%)**
DQN	64 × 64	35.09
Traditional machine learning	64 × 64	87.66
	128 × 128	62.83
YOLOv8n	64 × 64	99.42
	128 × 128	96.07
YOLOv8m	64 × 64	99.42
	128 × 128	91.86
YOLOv8l	64 × 64	97.13
	128 × 128	95.51
YOLOv8n-NWD	64 × 64	99.23
	128 × 128	95.25
YOLOv8m-NWD	64 × 64	99.04
	128 × 128	97.43
YOLOv8l-NWD	64 × 64	99.33
	128 × 128	96.10
Ours-YOLOv8n	64 × 64	99.61
	128 × 128	96.69

### 4.7 Visualization of test results

**Figure 6:**
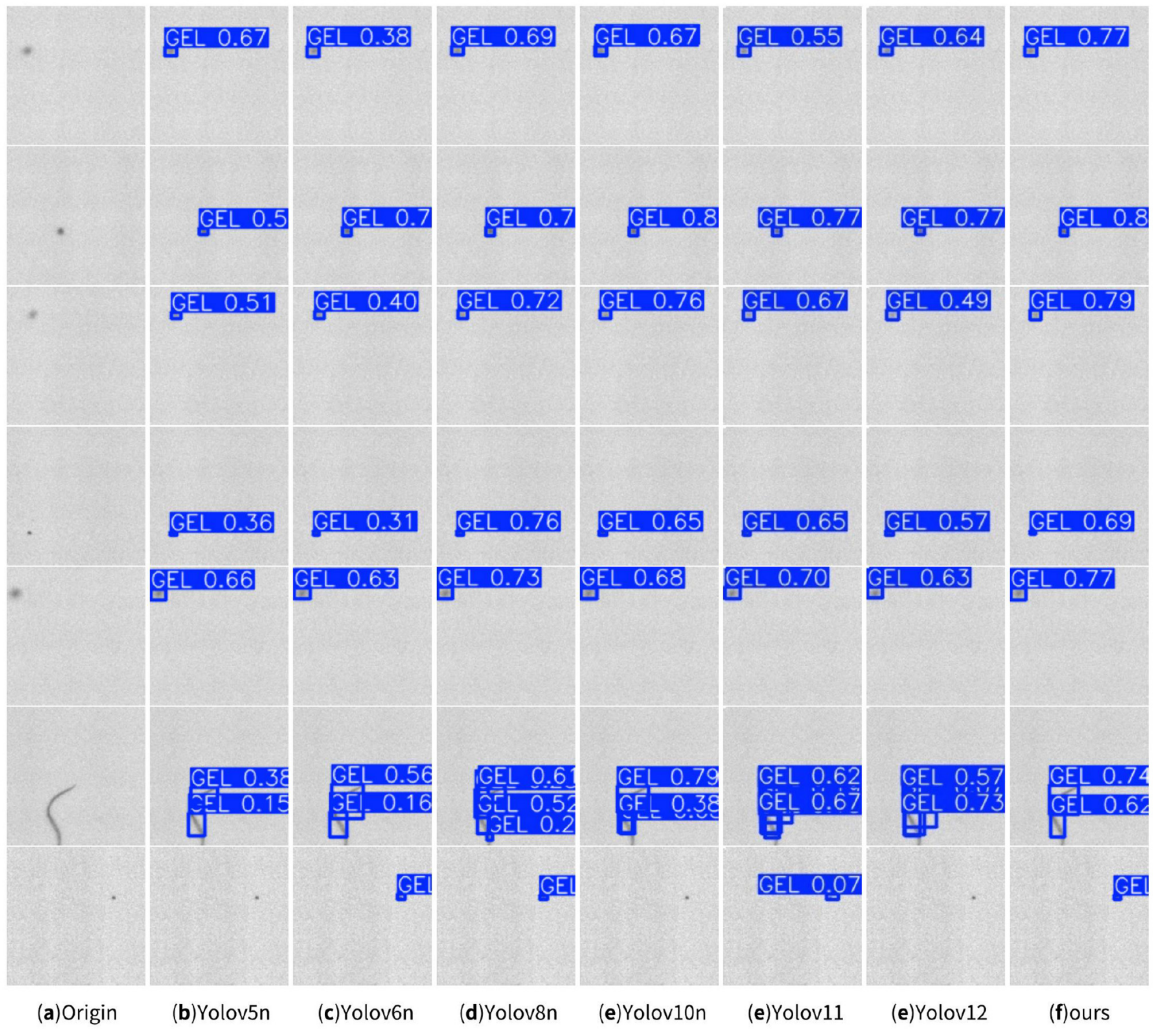
Visual analysis of detection results across YOLO algorithm variants.

Visualization of defect detection results across YOLO variants are shown in Figure 6. The enhanced YOLOv8n model (ours), integrating NWD, CBAM, and Mean Teacher, exhibits superior detection accuracy and recall compared to other YOLO models, effectively minimizing missed detections. This confirms the robustness of the proposed improvements for industrial defect inspection.

### 4.8 Generalization verification

Industrial environments are often complex, where lens contamination and illumination variations are common yet critical interference factors; during prolonged use, lenses are prone to accumulate contaminants such as dust and grease, leading to blurred captured images, while changes in lighting conditions—such as natural light fluctuations between sunny and cloudy weather or aging lighting equipment—alter image brightness and contrast, thereby degrading data quality and subsequent analysis. To validate the model's operational reliability under real-world conditions, experiments were conducted across diverse environments. This study adopts an adaptive blur and localized brightness adjustment method for data augmentation, applying randomized perturbations to input grayscale images to simulate complex detection environments and enhance the model's robustness to varying illumination and blur levels. The adaptive blur process randomly selects a blur kernel size (ensured to be odd) and applies Gaussian blur to simulate focal variations or motion blur. For localized brightness adjustment, a brightness scaling factor within the range [0.7, 1.5] is randomly selected to simulate illumination changes, and a randomly chosen rectangular region in the image undergoes pixel value scaling to brighten or darken the area, improving the model's adaptability to uneven lighting. Generalization validation results under varying environmental conditions are summarized in Table 9. The improved YOLOv8 model effectively enhances detection performance, particularly demonstrating higher precision and stability in handling complex scenarios and multi-target detection tasks.

**Table 9:** Performance comparison of different object detection models.

**Networks**	**Brightness adjustment**	**Adaptive blur process**	**mAP@0.5**	**Precision (%)**	**Recall (%)**
YOLOv8n-NWD	✓		85.23	85.52	79.56
		✓	84.69	84.37	78.79
			86.68	86.48	81.28
YOLOv8m-NWD	✓		88.60	87.30	84.63
		✓	83.81	83.03	77.61
			88.82	78.85	89.75
YOLOv8l-NWD	✓		88.94	84.60	87.78
		✓	87.55	85.90	83.04
			87.89	87.04	83.24
YOLOv8n-MeanTeacher	✓		83.82	85.57	75.32
		✓	81.80	74.31	82.22
			83.33	73.40	83.40
YOLOv8m-MeanTeacher	✓		83.75	80.46	80.53
		✓	83.14	75.43	83.50
			88.49	80.93	87.86
YOLOv8l-MeanTeacher	✓		86.38	79.11	85.96
		✓	83.05	73.15	84.58
			85.93	75.61	86.89
YOLOv8n-CBAM	✓		81.28	69.48	82.22
		✓	83.83	84.90	76.62
			87.59	85.25	85.25
YOLOv8m-CBAM	✓		89.44	86.78	85.75
		✓	84.35	77.38	82.89
			85.56	74.81	85.66
YOLOv8l-CBAM	✓		87.08	83.44	84.39
		✓	86.26	87.54	79.41
			85.51	81.08	83.62
Ours-YOLOv8n	✓		86.33	87.42	80.08
		✓	82.40	83.18	74.73
			87.87	89.38	80.21

## 5 Discussion

In industrial production, defect detection in polymer material films is highly challenging due to the extremely small size of detection targets and difficulties in image acquisition. Traditional machine learning-based inspection methods, which suffer from inefficiency, are no longer suitable for rapidly advancing industrial processes. This study proposes an improved YOLOv8 method that integrates the Normalized Wasserstein Distance (NWD) loss with the bounding box regression loss function, embeds the CBAM (Convolutional Block Attention Module) attention mechanism into high-level networks, and employs the Mean Teacher framework for semi-supervised learning. Experimental results demonstrate that integrating NWD, CBAM, and Mean Teacher into the YOLOv8n framework achieves an 8.26% improvement in mAP@0.5 compared to the baseline model without increasing model parameters, enhancing generalization capability and enabling more precise localization and detection of small defects on cast films. For real-world industrial scenarios, the detection rate is adopted to evaluate model performance. Results show that the improved YOLOv8 algorithm achieves detection rates exceeding 95.0% across defect images of varying sizes, meeting the stringent requirements for high-precision small-target defect detection in industrial production. In our study, we analyzed model failure cases focusing on false positives. False positives often result from environmental impurities, lighting-induced background fluctuations, and missed annotations during labeling. To mitigate this, we applied data augmentation and an attention mechanism to improve model robustness. Despite these efforts, eliminating false positives entirely remains challenging. Future work will explore more advanced techniques to enhance model accuracy and robustness.

## Data Availability

The raw data supporting the conclusions of this article will be made available by the authors, without undue reservation.
